# The Cambridge Executioner

**Published:** 1891-11-07

**Authors:** 


### The Doctor's Armchair.

### THE CAMBRIDGE EXECUTIONER.

The doctor of the present day is a fighting man. He is not willing any longer to play second fiddle to the man of mere words and ideas. He is in fine form for the intellectual tournament. He has been trained on the stiff battle-ground of science, and fed on an enduring diet of facts and realities. His intellectual muscles are firm, and his "wind" is perfect. His nerves are of iron, and his mental eye keen and quick. His whole attitude seems to say to the "word-spinner," "Come on: I am waiting for you."

The scientific doctor does not stand by himself: he is at the head of an army—an army that is destined to win. Ranked with him is the whole body of practical men of every profession and trade. The practical men invariably carry the day when fighting is to be done. They are like Cromwell's Ironsides: they cannot be conquered. Professor Jebb and his ghostly array of Grecians can no more stand against the forward movement of the scientific and practical world than a mad Captain Webb can shoot Niagara upwards. Sir George Humphry, the Cambridge professor of surgery, and a man of ripe years and mature wisdom, has issued a sober and gracious, but firmly-worded protest against the continuance of the compulsory study of Greek for all and sundry who may desire a Cambridge degree in arts. Sir George does not despise Greek; he does not even think little of it. On the contrary, he values it highly as a means of mental discipline and high culture. None of us wish to take away one iota from the honour and glory of Greek. So far from that, we admire with the profoundest admiration the man who has a mind of the peculiar order which loves to devote itself to pure ideas, and their expression in the purest forms. Greek is not merely a fine language. At the present day it is, so to speak, the living again of some of the freest, broadest, subtlest, and mentally purest of thinkers the world has ever seen. We grant to the Grecians at once that they are, or ought to be, intellectually fine fellows, and that they have in their specialty a polishing machine which ought to bring up their minds to the sharpest edge and the most glowing brilliancy. The present writer, though a doctor, knows something of Greek: he wishes he knew a very great deal more.

There is then no intention to disparage either Greek or Grecians, or to take away by one hair's-breadth the honour which is due to both. But when all is said and done, the world is still a practical world, and a university is an institution for the world, not for a limited part of it, even though that part be so noble as Greek and Grecians. The average middle-class Englishman values a university experience, and the middle-class Englishman of the present day values such an experience much more than his father and his grandfather did. Now that the average middle-class man has become comparatively wealthy he goes out into society a good deal, and he likes it. But he is often very painfully conscious of one drawback. When he meets a university man at dinner he becomes aware that such a man has an ease, a simplicity, and what may be called a cultured literary aptitude to which he himself is a stranger. Even the average doctor, who has had no university education, becomes subtly aware that his medical neighbour and friend, who has had the advantage of such an education, is his social and intellectual superior. This is a state of things which the proud gorge of the self-respecting Englishman rises at. Let us be thankful that it is so. A just self-respect and an adequate consciousness of personal dignity are among the surest of all guarantees of excellence of life and behaviour.

It is the object of universities to make men, and men of culture. Now, it cannot be denied that most kinds of study promote intellectual culture. Half-an-hour ago an aged engineer left the writer's consulting-room. He never saw the inside of a college, or even of a grammar school; he has been engaged in the construction and repair of ship's engines and boilers for sixty-five years. He is a man of fine and cultured tone of mind. An eminent mathematician, who in his Cambridge days was within an ace of being senior wrangler, and who knows the old gentleman intimately, but yesterday expressed his profound admiration for the general culture and intellectual capacity of this active minded engineer of eighty. Similarly, the writer knows several quite common working men, whose minds and characters have attained to a high degree of culture and grace from the study of the literature and morality of the Old and New Testaments. It is, indeed, manifest to all observant persons that many kinds of study tend to promote, not only intellectual culture, but intellectual culture of the very highest order.

Therefore, it is plain that all kinds of persons are capable of enjoying and profiting by university education and experience. It is equally plain that all kinds of persons are not capable of valuing and loving Greek. But neither are all kinds of persons capable of valuing and loving music, or mathematics, or physiology, or medicine. On what ground, then, of reason or of right does the Grecian claim to exclude from university education and experience those who have no taste for his peculiar specialty? The Grecian affirms that Greek is the finest of all the instruments of mental culture. But what is mental culture? Is it not mental breadth, mental clearness, mental judgment, and, above all, mental justice? Now, even for Grecians, "the proof of the pudding is in the eating." What sort of mental breadth, mental clearness, mental judgment, and mental justice did the Grecians show last week at Cambridge, when they decided by two votes to one to continue the regulation which insists that nobody shall claim the distinction of a Cambridge degree in arts who cannot show a smattering knowledge of the Greek language and literature? What the Grecians practically affirmed was this, that a limited acquaintance with the Greek Grammar, and the power to turn into English one or two Greek authors, with the further power of turning a few English sentences into not ungrammatical Greek, is more decided evidence of mental culture than an easy familiarity with mathematics, or sound scientific acquirements, or even a wide knowledge and appreciation of general literature. This is the judgment of the Grecians and the result of their Greek culture. Then, says the practical man, Greek culture is on its last legs; for if it can produce no better results than these, it is not worth preserving. Somebody has said that Greek is to be preserved as a "shibboleth" at Cambridge. We quite believe it. But what is the function of a "shibboleth?" It is now what it has always been—that of an executioner. What does Professor Jebb think of himself in his capacity of chief hangman at his university?

